# Porcine Kidney Organoids Derived from Naïve-like Embryonic Stem Cells

**DOI:** 10.3390/ijms25010682

**Published:** 2024-01-04

**Authors:** Meishuang Li, Xiyun Guo, Linxin Cheng, Hong Zhang, Meng Zhou, Manling Zhang, Zhibao Yin, Tianxu Guo, Lihua Zhao, Han Liu, Xiubin Liang, Rongfeng Li

**Affiliations:** 1 Jiangsu Key Laboratory of Xenotransplantation, Nanjing Medical University, Nanjing 211166, China; meishuang@njmu.edu.cn (M.L.); guoxiyun@stu.njmu.edu.cn (X.G.); chenglx@stu.njmu.edu.cn (L.C.); zhanghong@singleronbio.com (H.Z.); mengzh10@njmu.edu.cn (M.Z.); zhang_man_ling@126.com (M.Z.); yinzhibao@njmu.edu.cn (Z.Y.); guotianxu2021@stu.njmu.edu.cn (T.G.); zhaolihua@njmu.edu.cn (L.Z.); liuhan@stu.njmu.edu.cn (H.L.); 2 State Key Laboratory of Reproductive Medicine, Nanjing Medical University, Nanjing 211166, China; 3 Department of Pathophysiology, Nanjing Medical University, Nanjing 211166, China; liangxiubin@njmu.edu.cn; 4 Key Laboratory of Targeted Intervention of Cardiovascular Disease, Collaborative Innovation Center for Cardiovascular Disease Translational Medicine, Nanjing Medical University, Nanjing 211166, China

**Keywords:** pig, ESCs, kidney, organoid

## Abstract

The scarcity of donor kidneys greatly impacts the survival of patients with end-stage renal failure. Pigs are increasingly becoming potential organ donors but are limited by immunological rejection. Based on the human kidney organoid already established with the CHIR99021 and FGF9 induction strategy, we generated porcine kidney organoids from porcine naïve-like ESCs (nESCs). The derived porcine organoids had a tubule-like constructure and matrix components. The porcine organoids expressed renal markers including AQP1 (proximal tubule), WT1 and PODO (podocyte), and CD31 (vascular endothelial cells). These results imply that the organoids had developed the majority of the renal cell types and structures, including glomeruli and proximal tubules. The porcine organoids were also identified to have a dextran absorptive function. Importantly, porcine organoids have a certain abundance of vascular endothelial cells, which are the basis for investigating immune rejection. The derived porcine organoids might serve as materials for immunosuppressor screening for xenotransplantation.

## 1. Introduction

Organ transplantation is necessary for patients with irreversible organ failure; however, there is a worldwide shortage of donor organs [1]. Kidney organoids are regarded as a potential solution to the shortage of donors. The first human renal organoid was reported by Taguchi in 2014 [2]. He successfully differentiated iPSC into metanephric mesenchyme (MM) and induced it in a nephroid organoid with renal tubules and glomerulus. In 2015, Takasato established a differentiation strategy for both ureteric bud (UB) and MM, effectively generating the nephrons attaching to collecting tubes via the combined use of CHIR99021 and FGF9 for induction [3]. In 2019, Low reported a system for differentiating vascularized renal organoids [4]. The proportion of glomerular and renal tubule production is controlled by regulating the proportion of proximal and distal renal vesicles (RVs) at the late stage of differentiation, and the quantity of vascular endothelial growth factor A is managed as a result. Although a preliminary construction strategy for human renal organoids has been devised, the developed renal organoids are thus far not appropriate for clinical transplantation. The challenge lies in the absence of certain cell types. Furthermore, the lack of blood perfusion, immature vascular network, and off-target cell populations severely restrict the therapeutic potential of organoid transplantation.

Pigs are thought to be the potential donors for xenotransplantation since they closely resemble humans in terms of organ size, physiological function, and other aspects [5]. However, xenogeneic animal organ transplantation results in a more severe rejection response than human organs do, which is also accompanied by complicated side effects such as microthrombus development in the xenograft [6,7,8]. Xenotransplantation research has seen significant progress since the first GTKO pig was produced in 2002 by the knocking-out of the a1, 3-galactosyl transferase gene (*GGTA1*) to eliminate hyperacute immune rejection mediated by galactose-a1, 3-galactose (α-Gal) [9,10,11]. In 2016, the *GGTA1*, cytidine monophospho-N-acetylneuraminic acid hydroxylase gene (*CMAH*) and beta-1,4-N-acetyl-galactosaminyltransferase 2 gene (*B4GALNT2*) trigenic knocking-out pig were created and exhibited a significant reduction in immune rejection after xenotransplantation [12,13]. Meanwhile, human complement proteins CD46, CD55 or CD59 were integrated into a pig genome to eliminate the complement cascade and capillaries thrombus were eliminated by expressing human TBM or EPCR genes in the donor pig [14,15,16]. The primates which received the gene-edited pig kidneys and hearts had survived for the longest, at 499 and 195 days, respectively [17,18]. In 2021, two groups reported transplanting the 10 gene-edited pig kidneys into brain-dead patients and no acute immune rejection was found within 54 h after transplantation [19]. Almost at the same time, researchers transplanted the same genetically modified pig hearts into a heart failure patient, which prolonged his life for two months [20]. This milestone illustrates the enormous application future of gene-edited pig organs in clinical settings. The pig heart and kidney thus become the most promising organs for clinical applications [21,22,23]. And effective immunosuppressive medicines also become one of the crucial elements influencing these patients’ prognosis.

Although pig organs have a great deal of potential, this potential is nevertheless constrained by long breeding periods, expensive costs, and also the porcine endogenous retrovirus. If the porcine kidney organoid could be developed, it will become a good replicate of a pig organ for xenotransplantation, or, at least, could serve as a material for chronic immunosuppressive drug screening during organoid in vitro culture or after nonhuman primate in vivo transplantation. Regretfully, no reports on porcine organoid research have been found to date.

Porcine naïve ESCs have been effectively produced in several labs. Our lab also established porcine nESCs and identified them as possessing considerable pluripotency and differentiation capabilities [24]. In this study, the method of promoting the differentiation of porcine nESCs into kidney organoids in vitro was preliminarily investigated, which will become the foundation for the investigation of chronic immune suppressive medicines, and will ultimately promote kidney xenotransplantation.

## 2. Results

### 2.1. Determination of CHIR99021 Culture Duration for Porcine Renal Organoids Induction

The nESCs [24] established in our laboratory a couple of years ago were used for the differentiation of porcine kidney organoids.

**Figure 1:**
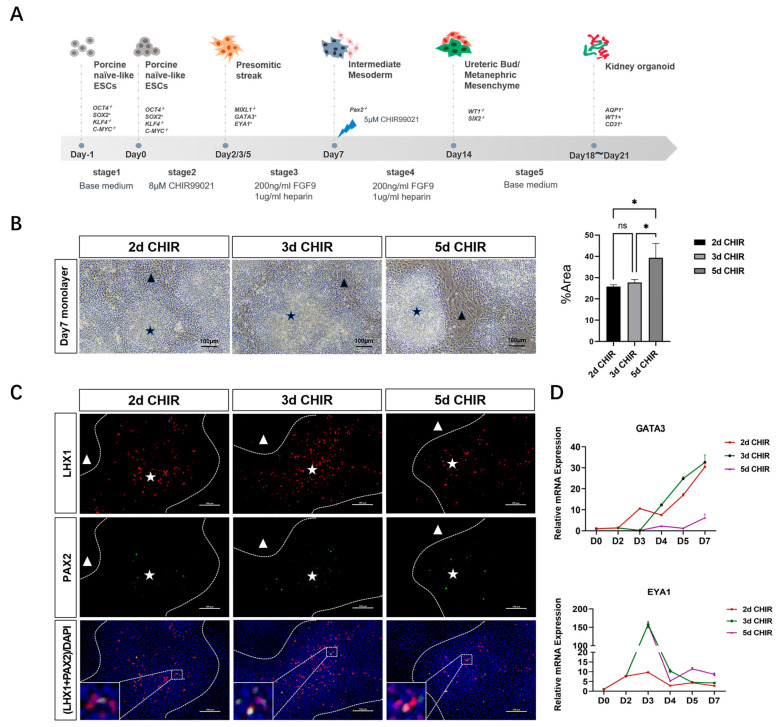
Determination of the treatment duration of CHIR for intermediate mesoderm induction. (**A**) Schematic diagram of porcine kidney organoids culture. (**B**) IM cell morphology on the seventh day: the black star shows colony-growth cells and the black triangle shows plane-grown cells (scale bar: 100 μm) (The “percentage area” in (**B**) represents the percentage of plane-grown cells area in the whole field). (**C**) The expression of anterior IM markers LHX1 and PAX2 of colony-growth cells was detected by immunofluorescence assay (The dotted line represents the boundary between colony-growth cells and plane-grown cells; the white star and white triangle show colony-growth cells and plane-grown cell, respectively; LHX1 and PAX2 were all localized in the nucleus) (scale bar: 100 μm). (**D**) The relative expression of anterior IM marker GATA3 and posterior IM marker EYA1 in different CHIR99021 cultures was detected by real-time PCR. * *p* < 0.05; ns, not significant.

According to former studies, the activation of the WNT signaling pathway can induce stem cells to transform into primordial stripe (PS) cells. The activation of the FGF signal pathway can induce the transformation of PS cells into intermediate mesoderm (IM) cells. IM cells contain two cell types, the anterior IM cells and the posterior IM cells. These two cell types will give rise to two key renal progenitor populations, MM cells and UB cells, and eventually form nephrons and collecting ducts, respectively. Related studies in human renal organoids suggest that the duration of WNT signal activation will affect the ratio of anterior IM cells and posterior IM cells, and ultimately affect the ratio of the nephron and collecting tube. In the differentiation scheme of porcine kidney organoids, we cultured stem cells with CHIR99021 for different durations (2, 3 or 5 days) to induce the activation of the WNT signaling pathway (Figure 1A). On day 7, an inspection of IM stage cells revealed two distinct cell arrangements: colony-growth cells and plane-grown cells (Figure 1B). An immunofluorescence assay showed that colony-growth cells expressed the anterior IM cell markers LHX1 and PAX2 (Figure 1C). Real-time PCR was used to detect the expression levels of anterior IM marker GATA3 and posterior IM marker EYA1 in different CHIR99021 application plans. The results showed that short-term CHIR99021 cultures (2 days) were more likely to induce the formation of anterior IM, while long-term CHIR99021 cultures (5 days) were more likely to induce the formation of posterior IM (Figure 1D). In order to attain both anterior and posterior IM at an appropriate ratio, we chose a 3-day CHIR incubation time to activate the WNT signaling pathway and equalize the ratio of anterior and posterior intermediate mesoderms in the next experiments.

### 2.2. Porcine Nephroid Organoids Contain Tubule-like Structures and the Majority of Renal Cell Types

**Figure 2:**
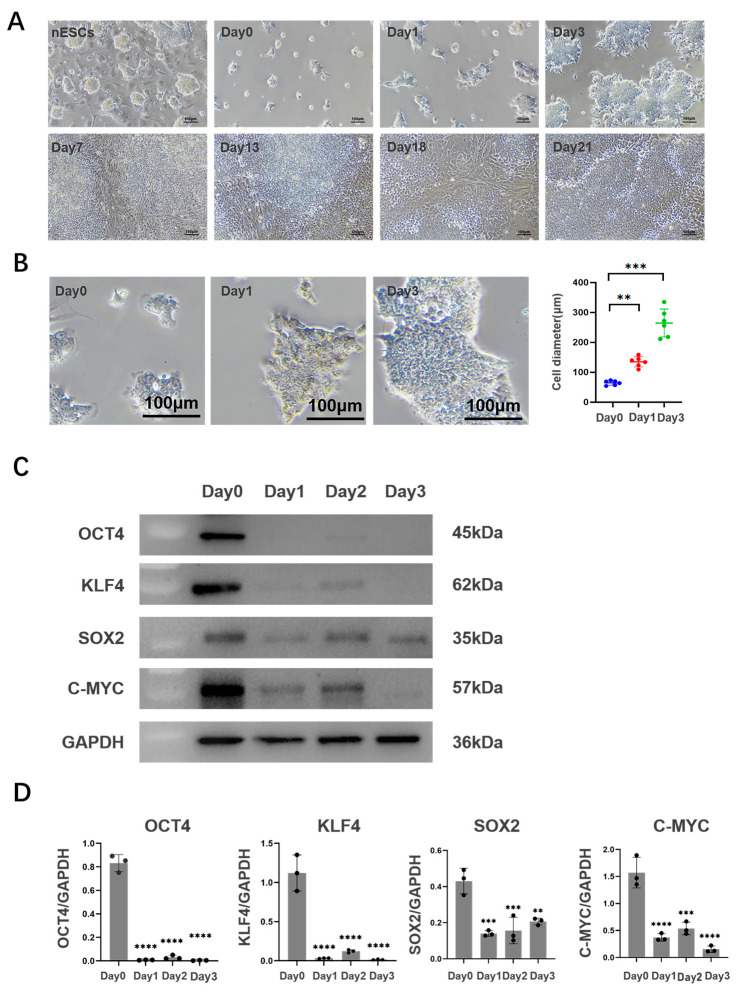
Characteristics of 2D culture cells. (**A**) Cell morphology of different days (scale bar: 100 μm). (**B**) Diameter contrast on the third day of differentiation (scale bar: 100 μm). (**C**,**D**) Expression of multipotent markers during the first three days after induced differentiation. The expression of multipotent markers OCT4, KLF4, SOX2, and C-MYC was detected by Western Blotting assay. ** *p* < 0.01; *** *p* < 0.001; **** *p* < 0.0001.

The 2D culture was attempted first and the morphological changes in porcine nESCs were observed (Figure 2A). It was found that nESCs lost their original characteristics after adding an inducer. On day 3 of induced differentiation, the cell density increased and the colony diameter grew dramatically and formed into a spiky shape (Figure 2B). On day 7 of induced differentiation, the cell density further increased and showed two mentioned different cell arrangements, as shown in Figure 1B. Then, colony-like cells protruded from the Petri dish’s surface gradually and the degree of cell convergence rose steadily, even on the 21st day after induction differentiation (Figure 2A). To demonstrate stem cell differentiation, we assessed the stemness of cell colonies formed on day 3 after differentiation via staining with the stemness markers OCT4, SOX2, KLF4, and C-YMC. Western Blotting and immunofluorescence experiments demonstrated that the expression of pluripotency markers in nESCs was decreased after the addition of inducible molecules (Figure 2C,D and Supplementary Figure S1).

**Figure 3:**
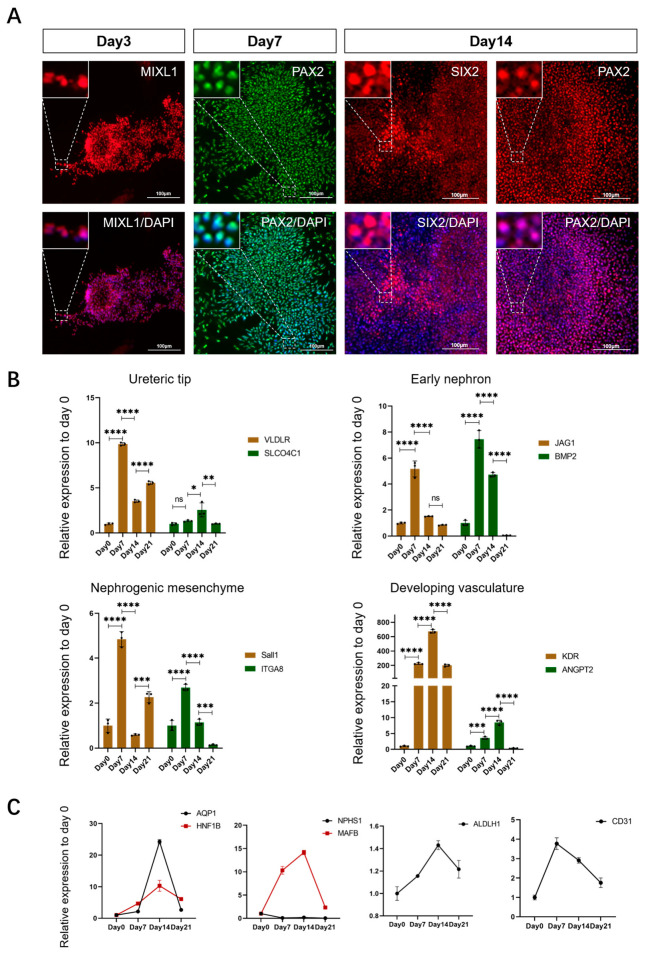
Changes in gene expression in 2D cultured porcine kidney organoids. (**A**) Expression of special markers in different periods. The specific markers for PS cells (MIXL1), IM cells (PAX2), MM cells (SIX2 and PAX2), and UE cells (SIX2 and PAX2) were discovered in the corresponding porcine kidney organoids on days 3, 7, and 14 of 2D culture by immunofluorescence test (MIXL1, PAX2, and SIX2 were all localized to the nucleus) (scale bar: 100 μm). (**B**) Expression level changes in renal progenitor cell markers at 4 time points (day 0, 7, 14, and 21) of the 2D cultured porcine kidney organoids. The relative expression levels of markers about early nephrons (JAG1, BMP2), nephrogenic stroma (SALL1, ITGA8), ureteral buds (VLDLR, SLCO4C1), and endothelial progenitor cells (KDR, ANGPT2) were measured by real-time PCR. (**C**) Expression level changes in mature nephron components markers at 4 time points (day 0, 7, 14, and 21) of the 2D cultured porcine kidney organoids. The relative expression levels of markers about podocytes (NPHS1, MAFB), proximal tubules (AQP1, HNF1B), distal tubules (ALDLH1), and endothelial cells (CD31) were measured by real-time PCR. Each value was averaged from three replicates and presented as mean ± SD. * *p* < 0.05; ** *p* < 0.01; *** *p* < 0.001; **** *p* < 0.0001; ns, not significant.

Previous studies have shown that, in the process of mammalian nephrogenesis, cells derived from the PS stage migrate to form the IM, which gives rise to the renal progenitor cell population (MM/UB) and eventually to the nephron and collecting duct. In our study, we found that, after 3, 7, and 14 days of 2D culture, the specific markers for PS cells, IM cells, MM cells, and UB cells were discovered in the corresponding induced cell mass (Figure 3A). These results indicated that the differentiation of porcine kidney organoids followed a similar pattern to embryonic nephrogenesis. Meanwhile, we detected the expression levels of the markers for renal progenitor cells including early nephrons, nephrogenic stroma, ureteral buds, and developing vasculature in the cultured cell cluster on different days (Figure 3B). In addition, the expression levels of the markers for mature renal cells including podocytes, proximal tubules, distal tubules, and endothelial cells were also measured (Figure 3C). The results showed that the progenitor cell number probably increased firstly and then decreased with the differentiation induction. The mature renal cell number probably also showed a surge and then a drop during the differentiation procedure. We speculated that, with the addition of the inducer, porcine nESCs gradually lose their stemness and grow toward kidney development. However, the expression of mature cells cannot always be maintained at a high level, which was considered to be due to the lack of cell–cell interaction in the 2D plane culture, thus limiting cell proliferation and migration. These problems might be solved via culturing porcine kidney organoids in a 3D environment.

**Figure 4:**
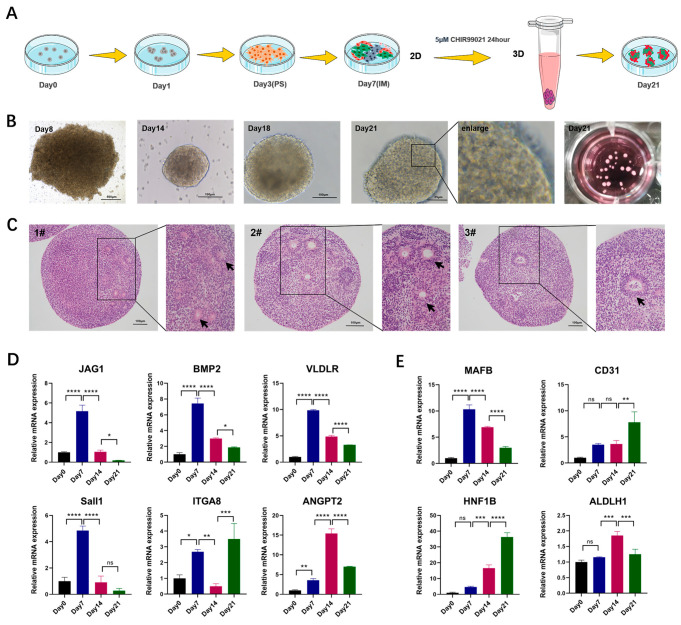
Generation and gene expression of porcine kidney organoids in 3D culturing environment. (**A**) Schematic diagram of 3D porcine kidney organoids culture. **(B**) Morphology of porcine kidney organoids on different culture days. (**C**) H&E staining of porcine kidney organoids (scale bar: 100 μm). (**D**) Expression level changes in renal progenitor cell markers at 4 time points (day 0, 7, 14, and 21) of the 3D porcine kidney organoids. The relative expression levels of markers of early nephrons (JAG1, BMP2), nephrogenic stroma (Sall1, ITGA8), ureteral buds (VLDLR), and endothelial progenitor cells (ANGPT2) were measured by real-time PCR. (**E**) Expression level changes in mature nephron components markers at 4 time points (day 0, 7, 14, and 21) of the 3D cultured porcine kidney organoids. The relative expression levels of markers about podocytes (MAFB), endothelial cells (CD31), proximal tubules (HNF1B), and distal tubules (ALDLH1) were measured by real-time PCR. Each value was averaged from three replicates and presented as mean ± SD. * *p* < 0.05; ** *p* < 0.01; *** *p* < 0.001; **** *p* < 0.0001; ns, not significant.

The 3D suspended porcine kidney organoids culture model is shown in Figure 4A. The mRNA was collected on day 21 and compared with the 2D cultured counterparts. The results showed that the 3D cultured organoids had a better maturity with the lower expression level of progenitor cell markers and higher expression level of mature cell markers (Supplementary Figure S2). The morphology of suspended organoids was checked on day 8, day 14, day 18, and day 21. It was found that the cell clusters cultured in 3D on day 8 were loose in structure. On day 14, the cell mass structure was dense, and some tubule-like structures appeared in the periphery. On day 21, multiple high-refraction tubule-like structure contours could be observed at the edges of organoids (Figure 4B). HE staining showed that there were matrix components with obvious eosin staining, as well as the multiple circular structures, which might be derived from the cross sections of the tubule-like structures in the organoids. (Figure 4C).

**Figure 5:**
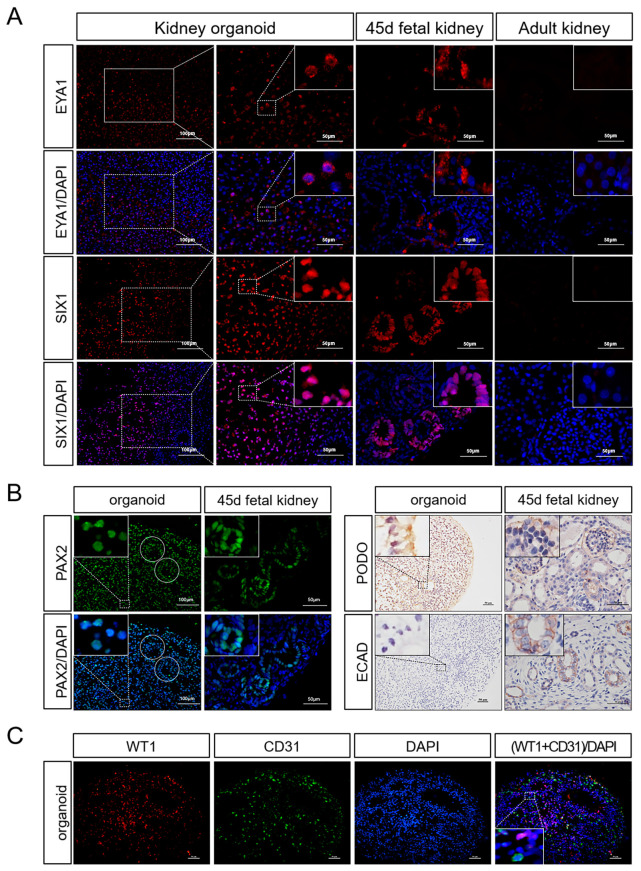
Gene expression in 3D porcine kidney organoids. (**A**) Expression of renal progenitor cell markers in the organoids on day 21 and fetal porcine kidney on day 45. Renal progenitor cells markers (EYA1, SIX1) were measured by immunofluorescence test (EYA1 was localized in the cytoplasm, SIX1 was localized in the nucleus). (**B**) Expression of mature renal cell markers in the organoids on day 21 and fetal porcine kidney on day 45. Mature nephron components markers (PAX2, E-CAD, PODO) were measured by immunofluorescence test and immunohistochemistry test (the circular dotted lines are tubule-like structures; PAX2 was localized in the nucleus; and PODO and ECAD were localized to the cell membrane). (**C**) Whole-mount co-staining of the organoids for WT1 and CD31 on day 21 (WT1 was localized in the nucleus and CD31 was localized to the cell membrane) (scale bar: 50 μm).

**Figure 6:**
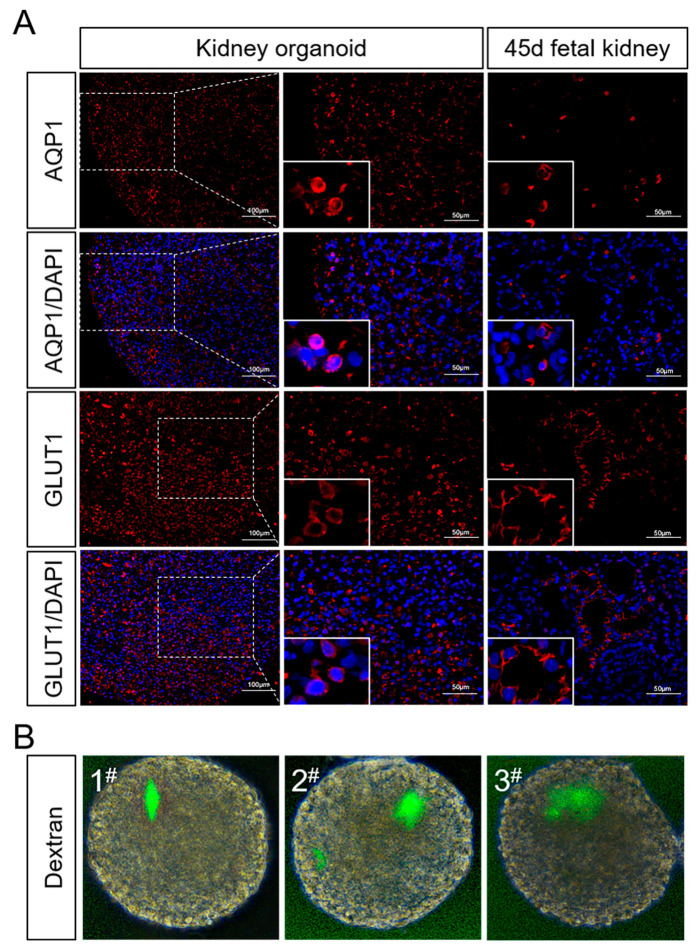
Identification of partial functions of porcine kidney organoids. (**A**) The expression of AQP1 and GLUT1 in the organoids on day 21 and fetal porcine kidney on day 45 were measured by immunofluorescence test (AQP1 and GLUT1 were localized in cytoplasm) (scale bar: 50 μm). (**B**) Live images of porcine kidney organoids on day 21 incubated with fluorescence-labeled dextran.

We also collected the organoid mRNA on day 7, 14, and 21 of differentiation to detect the changes in the expression levels of markers in the process of nephrogenesis. The results showed that the markers for early nephrons, nephrogenic stroma, ureteral buds and vascular progenitor cells all showed elevated expression during the pre-differentiation process. With the growth of organoids, the expression of these progenitor cell markers gradually decreased (Figure 4D). Compared with day 0, marker expression levels in the mature nephron including the proximal tubule, distal tubule, podocyte, and vascular endothelial cells were significantly increased on day 21 using real-time PCR (Figure 4E). EYA1 and SIX1, the progenitor cell markers, were still expressed in some organoids, according to immunofluorescence staining (Figure 5A). In addition, markers for tubular structures (PAX2), podocytes (WT1, PODO), and endothelial cells (CD31) could also be detected (Figure 5B,C). Meanwhile, we discovered that proximal tubule markers (AQP1) had higher expression levels, but distal tubule and collecting tubule markers (E-CAD) were hardly expressed (Figure 5B and Figure 6A). This indicated that porcine kidney organoids expressed PAX2, WT1, CD31, PODO, and AQP1, but did not express E-CAD, implying that the organoids we generated included the majority of the cellular compounds contained in normal kidneys.

### 2.3. Porcine Kidney Organoids Obtain Dextran Uptake Functions

The expression of proximal tubule functional proteins was detected by an immunofluorescence test of porcine kidney organoids on day 21. The results showed that some cells of porcine organoids expressed aquaporin1 (AQP1) and glucose transporter1 (GLUT1) (Figure 6A). To further demonstrate the absorptive function of porcine kidney organoids, in vitro dextran uptake assay was used to examine the absorptive capacity of proximal tubules of organoids. The results showed that dextran of 10,000 kDa could be absorbed by kidney organoids (Figure 6B).

### 2.4. Porcine Kidney Organoids Show Cell Apoptosis and Proliferation

**Figure 7:**
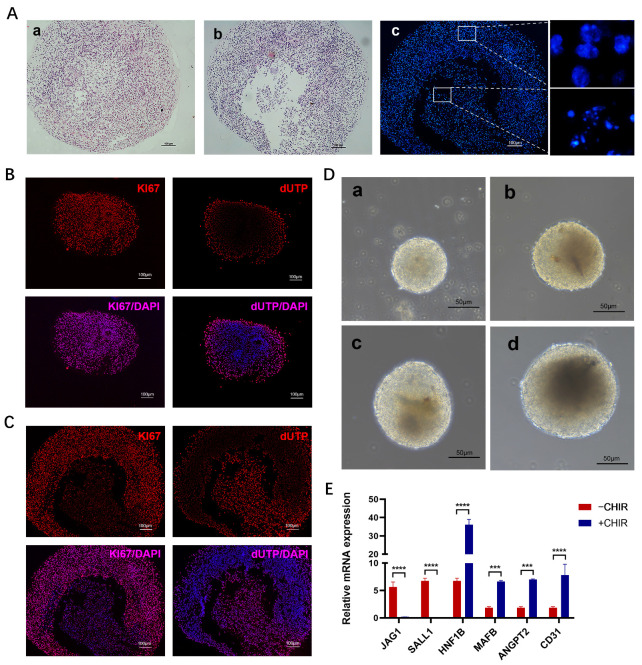
Apoptosis and proliferation of porcine kidney organoids and effect of CHIR pulse on the expression of organoid genes expression levels on day 21. (**A**) a: HE staining of the peripheral parts of porcine kidney organoids. b: HE staining of the core parts of porcine kidney organoids. c: DAPI staining of the nucleus. (**B**) Proliferation (KI67) and apoptosis (dUTP) in peripheral region of the organoids on day 21 were measured by immunofluorescence test. (**C**) Proliferation (KI67) and apoptosis (dUTP) in core region of the organoids on day 21 were measured by immunofluorescence test (KI67 and dUTP were localized in the nucleus). (**D**) Morphology of porcine kidney organoids on day 21 with different diameters. (**E**) The relative expression levels of markers of nephron progenitor cells (JAG1, SALL1, ANGPT2) and mature nephron components (HNF1B, MAFB, CD31). Each value was averaged from three replicates and presented as mean ± SD. *** *p* < 0.001; **** *p* < 0.0001.

We examined the apoptosis and proliferation of porcine kidney organoids on day 21. HE staining showed that, in the peripheral parts of porcine kidney organoids, the organoids were still completely spherical, while, in the core parts of porcine kidney organoids, there was a significant loss of central cells (Figure 7A). This can be proved by the DAPI staining of the nucleus. The peripheral cell nucleus was intact, while the central cell nucleus showed nuclear pyknosis, nuclear fragmentation, and other manifestations of apoptosis (Figure 7A). In addition, immunofluorescence showed that organoid division and proliferation mainly concentrated in the peripheral region and the core region showed significant apoptosis (Figure 7B,C).

To further optimize the cultivation conditions of kidney organoids, we classified the obtained organoids according to their diameters. Based on the results of the immunofluorescent staining (Figure 7B,C) and morphological observation (Figure 7D) of organoids with different diameters, we speculated that, with the increase in kidney organoid diameter, the number of undifferentiated cells or apoptotic cells in the central dark region gradually increased.

### 2.5. CHIR99021 Pulse Induces Better Maturation of Kidney Organoids

In our study, we used CHIR99021 stimulation for 24 h after 3D sphere formation to briefly activate WNT signaling (CHIR99021 pulse). We compared the gene expression of organoids created following 3D sphere aggregation with or without CHIR99021 pulse in order to determine the impact of CHIR99021 pulse on the development of porcine kidney organoids. The results showed that the expression levels of early renal markers JAG1 and SALL1 were significantly lower, whereas the expression levels of the vascular progenitor cell marker ANGPT2, as well as the mature cell markers HNF1B, MAFB, and CD31, increased more with CHIR99021 pulse than without CHIR99021 pulse (Figure 7E).

## 3. Discussion

In this study, porcine naïve-like ESCs were successfully differentiated into porcine kidney organoids by referring to the culture scheme of human kidney organoids and modifying the culture medium accordingly [3]. These organoids were demonstrated to have similar cellular composition, gene expression, and dextran reabsorption capacity, to some extent, to kidneys.

The cultivation strategy for human kidney organoids has been gradually enhanced as a result of optimization by different research groups [3,25,26]. However, the application of human kidney organoids is mostly focused on the development of disease models or as individualized models for screening nephrotoxic medicines [27,28,29]. Organoids allow researchers to make autologous or human lymphocyte antigen-compatible tissue for regenerative medicine, but their clinical translation into cell replacement treatment is still in its early stages [30]. The goal of regenerative medicine is to fully restore or reestablish the structure and function of injured human cells, tissues, and organs by replacing or recreating them. The absence of cellular components or the lack of vascularization in the current human organoids greatly limits their safety and effectiveness in regenerative medicine [4,31]. Porcine organs are thought to be an excellent donor for regenerative medicine organ transplantation. However, xenoimmune rejection has seriously hindered the progress of xenotransplantation. The development of a porcine kidney organoid model and its application in the study of immune rejection in xenotransplantation, preliminary drug screening, or drug toxicity research has a promising future.

In our study, we firstly identified an appropriate medium for the induction and differentiation of porcine kidney organoids and proved that the treatment time and duration of CHIR99021 determines the activation of the WNT signaling pathway and ultimately affects the differentiation of intermediate mesoderm, which is consistent with studies of human kidney organoids [3,32,33]. Early researchers have proposed that in vitro 3D models provide better access to natural physiological conditions than traditional 2D models because they form structures with specific polarities and increase cell communication [34]. In this study, the differences between 2D planar culture and 3D suspension culture were further compared. Lower gene expression levels of progenitor cells and higher gene expression levels of mature cells demonstrated that the 3D suspension culture resulted in a higher maturity of porcine nephroid organoids at day 21. Previous studies have shown that the kidney goes through PS, IM, and MM/UB stages during development [35]. By detecting the gene expression of the cell masses at different stages, including *Mixl1*, *Pax2*, *Six2*, and other genes, we believe that the differentiation process of porcine kidney organoids has a similar molecular biology pattern to that of normal nephrogenesis in embryo, although the organoids contain only tubule-like structures. Furthermore, porcine kidney organoids have been demonstrated to contain a variety of renal cell types according to the expression of tubular markers (PAX2), glomerular markers (PODO, WT1), proximal tubule indicators (AQP1), and vascular endothelial cell markers (CD31). In vivo, proximal epithelial cells reabsorb filtered glucose, amino acids, phosphates, and urea from the glomerular filtrate via renal specific transporters [3,36]. Our porcine kidney organoids have been shown to transport glucose into organoid lumens. During normal kidney development, WNT9b generated by the ureteral epithelium can stimulate nephron production in the metanephric mesenchyme. This was also confirmed in our work, where we found more mature renal organoids after adding the WNT signaling activator CHIR99021, indicating that the temporary activation of WNT signaling is important for organogenesis in porcine kidney organoids.

We referred to Takasato’s induction strategy with the combination of CHIR99021 and FGF9 to generate a porcine kidney organoid. The human kidney organoids they generated have a nephron and a collection tube, showing the tissue complexity and high degree of functionalization of the organoids [3]. The human kidney organoids produced by Low through regulating the quantity of vascular endothelial growth factor A have a more comprehensive vascular network [4]. Similarly, the porcine kidney organoids established in this study were also comparable in terms of cellular structure composition, tubule-like structures, and glucan uptake ability. However, the porcine kidney organoids were deficient in functional renal tubular maturity and glomerular angiogenesis compared with the abovementioned human kidney organoids. The poor maturity of porcine kidney organoids might be a result of improper induction techniques and/or the insufficient pluripotency of naïve-like ESCs. The use of fluid management devices in culture in vitro or the transplanting of kidney organoids into a renal capsule of recipient immunocompromised mice for in vivo maturation have been reported to increase the organoids’ degree of vascularization [37,38]. In order to improve the overall structural and functional maturity of porcine kidney organoids, we plan to modify the induction protocol (such as adjusting the CHIR99021 pulse duration or using Activin A) and/or apply the induced expanded pluripotent stem cells (EPSCs) that were established recently in our lab and have higher pluripotency as the initial cells for kidney organoid creation [25,39,40,41].

The use of porcine kidney organoids as a new model for xenograft-related immunosuppressive drug screening is based on the fact that porcine organoids can trigger the immune responses of the recipient to xenoantigens [42]. When hyperacute immune rejection occurs, vascular endothelial cells are recognized and activate the complementary regulatory system, resulting in thrombosis and platelet aggregation [43]. Acute immune rejection results in the activation of calcium-based vascular endothelial cells. The porcine kidney organoids we obtained have a certain abundance of endothelial cells, so we believe that they have the potential to be developed as a new model for the preclinical screening of immune-related inhibitors.

## 4. Materials and Methods

### 4.1. Cell Culture and Differentiation

Two naïve-like ESCs (nESCs) lines were used in this study. These cell lines were established by our lab previously and were identified to differentiate into 3 germ layers in vitro, with even neural and kidney precursors under defined conditions. The nESCs were cultured on MEF cells in LBX medium [24]: knockout serum replacement (KOSR) medium and N2B27 medium supplemented with 16 ng/mL FGF (Invitrogen, Carlsbad, CA, USA), 10 ng/mL hLIF (Millipore, Billerica, MA, USA), 1 μM PD0325901 (Sigma, Darmstadt, Germany), 3 μM CHIR99021(Sigma), 2 μM SB431542 (Tocris, Bristol, UK), and 2 mg/mL doxycycline (DOX, Tocris). Cells were grown under controlled conditions (38.5 °C; 95% air, 5% CO_2_). All experiments used porcine naïve-like ESCs (nESCs) as established previously. The nESCs were cultured on the feeder layer of mitomycin-treated ICR mouse embryonic fibroblasts. When the nESCs had converged to 70%~80%, they were plated on a Matrigel-coated culture plate at a rate of 15,000 cells per cm^2^. The next day, cells were treated with 8 μM CHIR99021 in LBX medium, as described previously, for 2 to 5 days, and then 200 ng/mL FGF9 and 1 μg/mL heparin were given for 5 to 2 days. The medium was changed every two days. On day 7, cells were treated with 5 μM CHIR99021 in LBX basal medium for 24 h (CHIR99021 pulse). The next day, the culture medium was replaced with an LBX medium containing 200 ng/mL FGF9 and 1 μg/mL heparin for another 5 days. Lastly, the pellets were cultured with LBX basal medium for 7~11 days. The medium was changed every two days. For 2D cultures, cells were kept growing in the inner plane of the dish until day 21. For 3D cultures, the differentiated cells were treated with 0.05% trypsin and centrifuged at 1300 rpm for 3 min to form pellets at the bottom of the Eppendorf, and then the pellets were transferred to the culture plate on day 7. The suspension culture was then maintained until day 21.

### 4.2. Immunocytochemistry

For immunostaining, cells were washed with PBS and fixed with 4% paraformaldehyde (Solarbio, Beijing, China) for 10 min, then permeabilized with 0.2%Triton X-100 in PBS for 10 min and blocked with 5%BSA at room temperature for 1 h. The cells were incubated with primary antibodies overnight at 4 °C, followed by incubation with secondary antibodies for 1 h at room temperature. The primary antibodies included OCT4 (Santa Cruz Biotechnology, Dallas, TX, USA), SOX2 (Cell Signaling Technology, Danvers, MA, USA), KLF4 (Cell Signaling Technology), C-MYC (Cell Signaling Technology), LHX1 (GeneTex, Alton PkwyIrvine, CA, USA), MIXL1 (proteintech, Chicago, IL, USA), PAX2 (Santa Cruz Biotechnology), and SIX2 (Abcam, Waltham, MA, USA). Secondary antibodies included goat anti-rabbit IgG Alexa Fluor 546 (ABclonal, Wuhan, China), goat anti-rabbit IgG Alexa Fluor 488 (Thermo Fisher Scientific, Waltham, MA, USA), goat anti-mouse IgG Alexa Fluor 546 (Thermo Fisher Scientific), and goat anti-mouse IgG Alexa Fluor 488 (Thermo Fisher Scientific). The nucleus was stained with DAPI.

### 4.3. Immunofluorescence and Immunohistochemical

The organoids were fixed with 4% paraformaldehyde overnight at 4 °C, and then they were embedded with 3% low melting point agarose. Finally, they were embedded with paraffin wax and sliced into 5 um thicknesses. The sections were dewaxed, rehydrated, and mended with antigen before being treated with the primary antibody at 4 °C overnight. The second antibody was then incubated for one hour at room temperature after being washed with PBS. The primary antibodies included the following: EYA1 (Abcam), SIX1 (Proteintech), PAX2 (Santa Cruz Biotechnology), WT1 (Abcam), E-CAD (Abcam), PODO (Absin), CD31 (Santa Cruz Biotechnology), AQP1 (Proteintech), GLUT1 (proteintech), and KI67 (Abcam); the secondary antibodies included goat anti-rabbit IgG Alexa Fluor 546 (ABclonal), goat anti-rabbit IgG Alexa Fluor 488 (Thermo Fisher Scientific), goat anti-mouse IgG Alexa Fluor 546 (Thermo Fisher Scientific), goat anti-mouse IgG Alexa Fluor 488 (Thermo Fisher Scientific), goat anti-rabbit IgG H&L(HRP) (Abcam), and goat anti-mouse IgG(H + L) (KeyGEN BioTECH, Nanjing, China). The nucleus was stained with DAPI.

### 4.4. Gene Expression Analysis

Total RNA was obtained from cells using Total RNA Kit (Promega, Madison, WI, USA) and cDNA was obtained from 1000 ng total RNA using HiScriptII Q RT Super Mix (Vazyme, Nanjing, China). Real-time PCR was performed with ChamQ SYBR qPCR Master Mix (Vazyme) by Real-Time System (Applied Biosystems, Waltham, MA, USA). All data were normalized to GAPDH expression and analyzed using the ΔΔCt method. The primers used are listed in Table 1.

### 4.5. Western Blotting

Cell proteins were obtained by lysis using RIPA buffers supplemented with protease inhibitors. Protein contents were determined using PierceTM BCA protein assay (Thermo Fisher Scientific), fractionated by SDS-polyacrylamide gels, and finally transferred to PVDF membranes. The membranes were blocked with 5% nonfat milk at room temperature for 2 h and treated with primary antibody at 4 °C overnight. After washing with TBST, the second antibody was incubated for 2 h at room temperature. The primary antibodies included the following: OCT4 (1:1000, Santa Cruz Biotechnology), SOX2 (1:1000, Cell Signaling Technology), KLF4(1:1000, Cell Signaling Technology), C-MYC (1:1000, Cell Signaling Technology), and GAPDH (1:10,000, ABclonal); the second antibodies included goat anti-rabbit IgG (1:10,000, Abcam). After Western blotting, the membranes were incubated with ECL and exposed to the ChemiDoc XRS+ gel documentation system (Bio-Rad Laboratories, Hercules, CA, USA). The samples were normalized to GAPDH expression and the density of the bands was analyzed using Image J software (version 1.8.0_112, National Institutes of Health, Bethesda, MD, USA).

### 4.6. Dextran Uptake Assay

Kidney organoids were incubated with 100 μg/mL fluorescent-labeled dextran (RHAWN, Shanghai, China) for 24 h. Then, the kidney organoids were transferred to a fresh medium and images of live cell culture were captured using a fluorescence microscope (Nikon MODEL ECLIPSE Ti2-U, Nikon Corporation, Tokyo, Japan).

### 4.7. Statistical Analysis

All data in this experiment were expressed as mean ± standard deviation (X ± S) and GraphPad software (version 8, San Diego, CA, USA) was used for significance analysis. A *t*-test was used to compare the two independent samples and *p* ≤ 0.05 was considered statistically significant. In all graphs, *p* ≤ 0.05 was marked as *, *p* ≤ 0.01 was marked as **, *p* ≤ 0.001 was marked as ***, *p* ≤ 0.0001 was marked as ****, and *n* ≥ 3; ns, not significant.

## Figures and Tables

**Table 1:** The primer sequence of real-time PCR.

Genes	Sequence (5′-3′)
GATA3	**F:** AACCACGTCCCGTCCTACTA **R:** GGTGGATGGACGTCTTGGAG
EYA1	**F:** CCCGGGGGATAAATGGACAG **R:** GTGCCATTGGGAGTCATGGA
VLDLR	**F:** CGGGAGTGCCAAAGGATCAA **R:** TCAGGTAGCCACCTACTGCT
SLCO4C1	**F:** ATGGAGGTTCTCAGGCTAAGG **R:** GCTGTGGTGTAGGTTGAAGAC
JAG1	**F:** CCTCCAACGACACGCCAGAAG **R:** TTGCCTCCTGACTGACTCTTGC
BMP2	**F:** CAGCTTCCACCACGAAGAATC **R:** CTGTGTCTGCTCCCGAAAGA
SALL1	**F:** TCCTCACCAGCTTTCGCAAT **R:** CGGTGACATTTGGTGGCTTG
ITGA8	**F:** GCACCACCTCACCTACAC **R:** CCCACACCCATTTCAAAGC
KDR	**F:** CGGACTCTCTCTGCCTACCT **R:** ACTGTTCTGCAGATACTGACTTAGA
ANGPT2	**F:** GGCTGGGAAATGAGTTTGTGT **R:** CCTCCTGTGAGCATCTGTGAA
AQP1	**F:** ATCATTGCCCAGTGTGTGGG **R:** GTGCCAATGATCTCGATGCC
HNF1B	**F:** TCTCAGTCTCAGGAGGAGGTT **R:** GAGGAGTTGAGGCTTTGTGC
NPHS1	**F:** TTGGGACAAGGAGGGGGAGAGG **R:** GCAGGGTTGGCAGACACGGACA
MAFB	**F:** CCTGGAGAATGAGAAGACAC **R:** CGGAGTTGGCGAGTTTC
ALDH1L1	**F:** CCTCATCATCTTCTCCGACT **R:** TTCATCTTCCCCACCTCTTC
CD31	**F:** AAGGAAGTGACCTTCTGGCG **R:** TTCCTCCACTGGGGCTATCA

**F:** Forward primer; **R:** Reverse *primer.*

## Data Availability

Data are contained within the article and Supplementary Materials.

## Supplementary Materials

The following supporting information can be downloaded at: https://www.mdpi.com/article/10.3390/ijms25010682/s1.
