# Supplementary material for: Porcine Kidney Organoids Derived from Naïve-like Embryonic Stem Cells
Source: Int J Mol Sci. 2024 Jan 4;25(1):682. doi: 10.3390/ijms25010682 (PMC10779635; doi:10.3390/ijms25010682)
Supplement: Supplementary file 1 [file ijms-25-00682-s001.zip › ijms-2750484-supplementary.pdf]

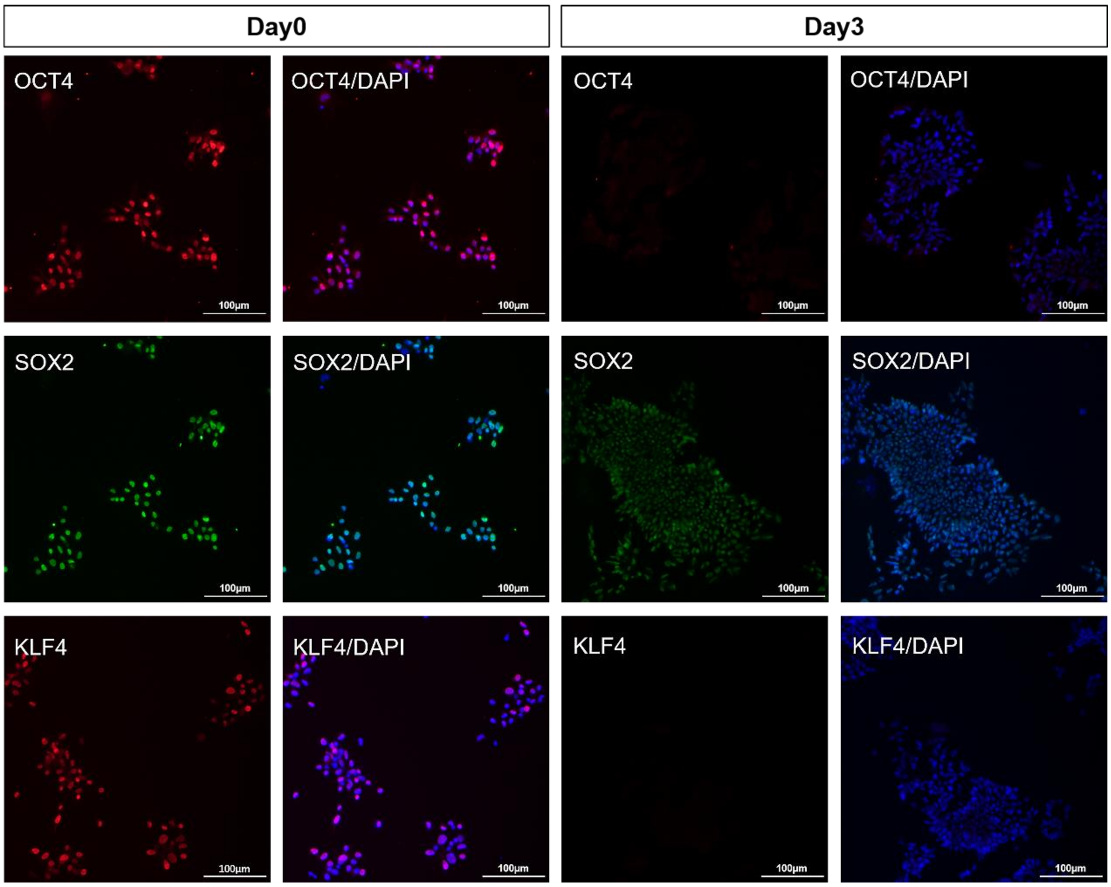

Figure S1. The expression of pluripotency markers was decreased in nESCs after induction. The expression of multipotent markers OCT4, SOX2, KLF4 of cell colonies on day 0 and day 3 were detected by immunofluorescence assay (scale bar:100μm).

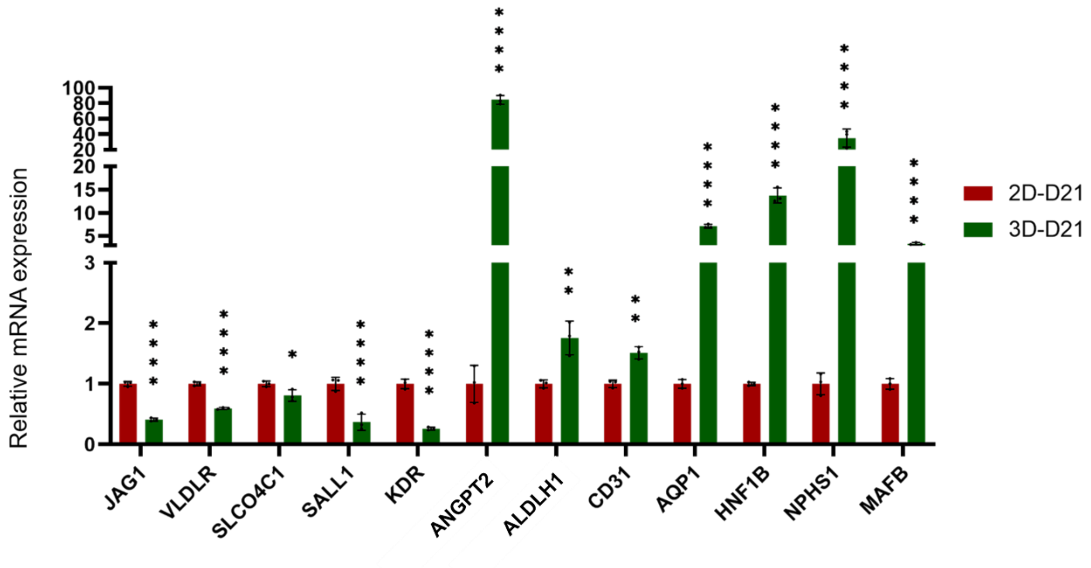

Figure S2. Comparison of related gene expression levels between 2D culture and 3D culture on day 21. The relative expression level of markers of nephron progenitor cells (JAG1, VLDLR, SLCO4C1, SALL1, KDR, ANGPT2) and mature nephron components (ALDLH1, CD31, AQP1, HNF1B, NPHS1, MAFB) were measured by Real-time PCR.

These un-cropped Western Blot membranes have been labeled with molecular weights, target protein and they belong to figure 2C in new version.

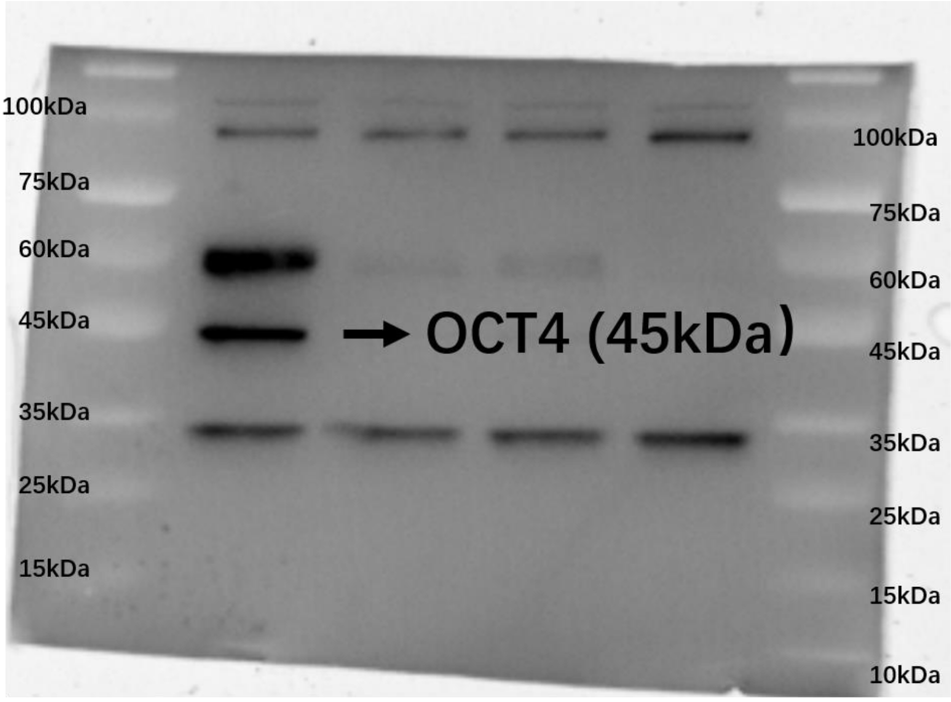

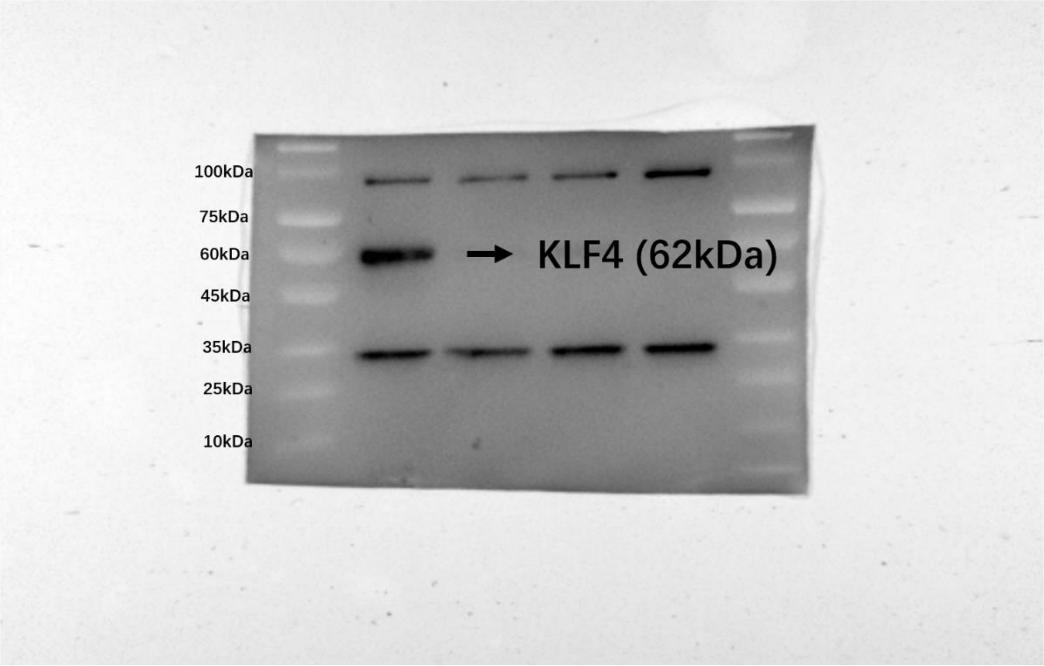

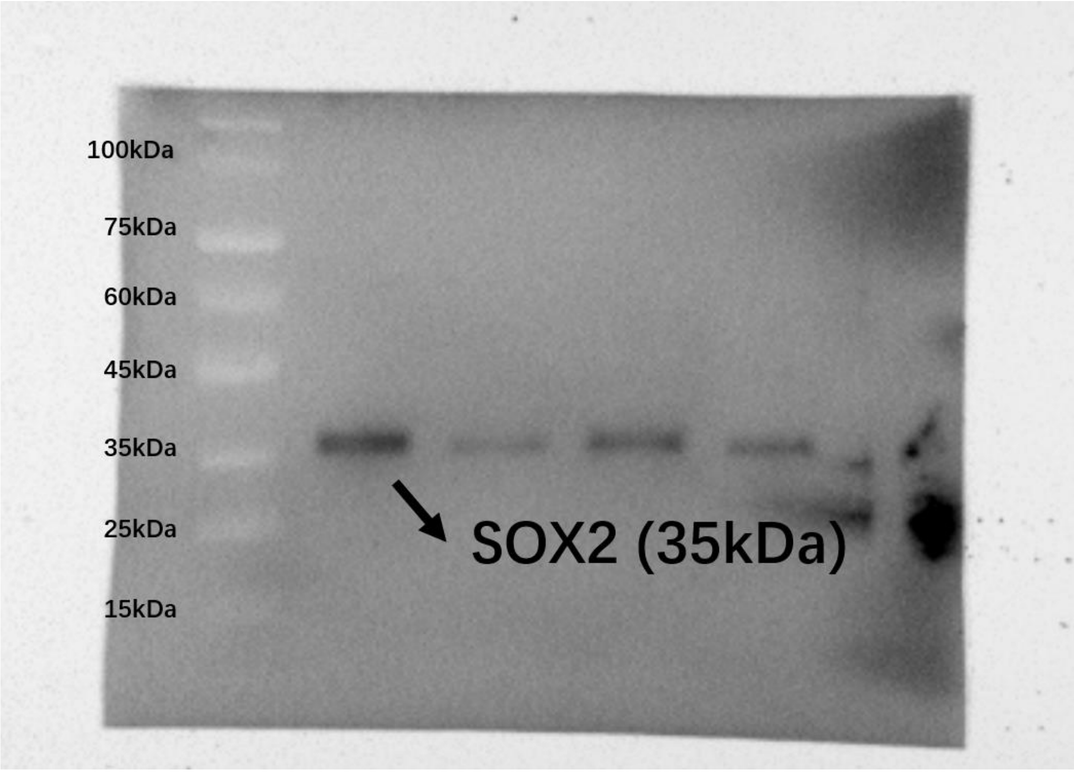
100kDa
75kDa
60kDa
45kDa
35kDa
25kDa
15kDa
SOX2 (35kDa)

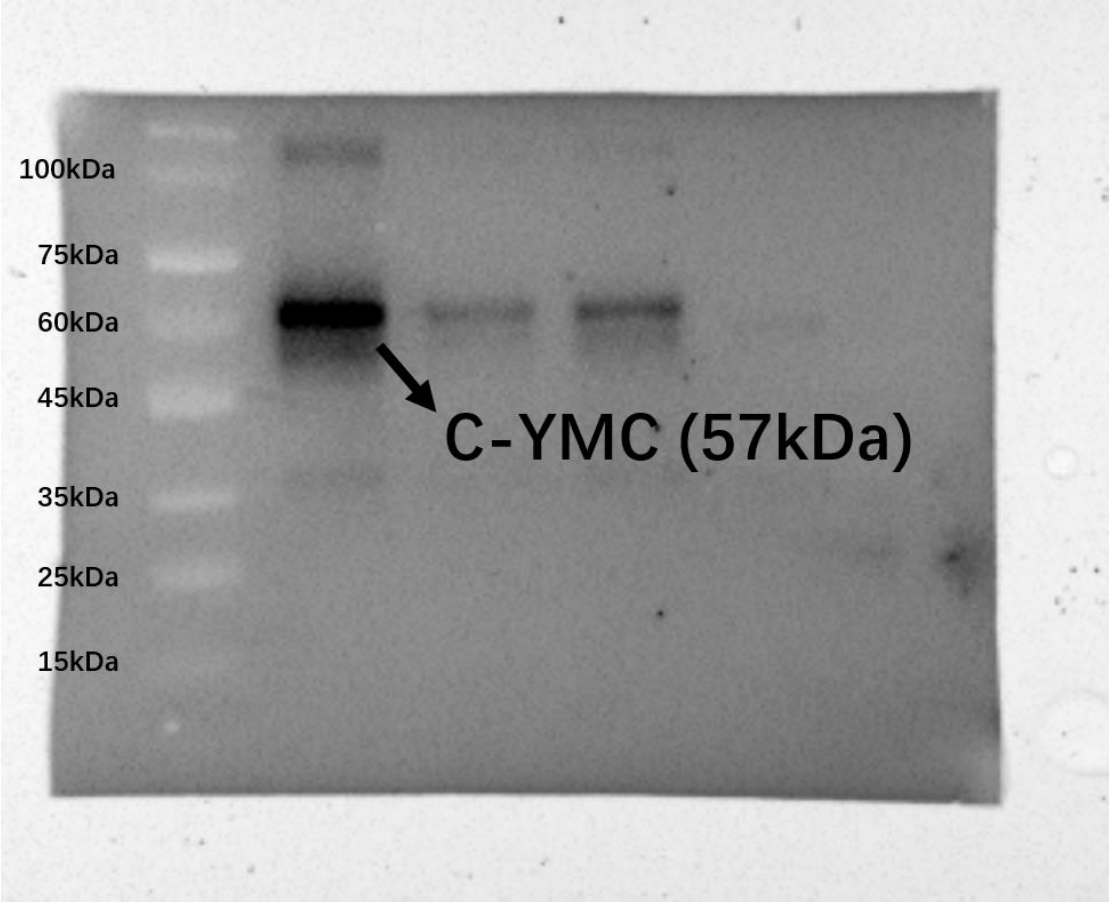
100kDa
75kDa
60kDa
45kDa
35kDa
25kDa
15kDa
C-YMC (57kDa)

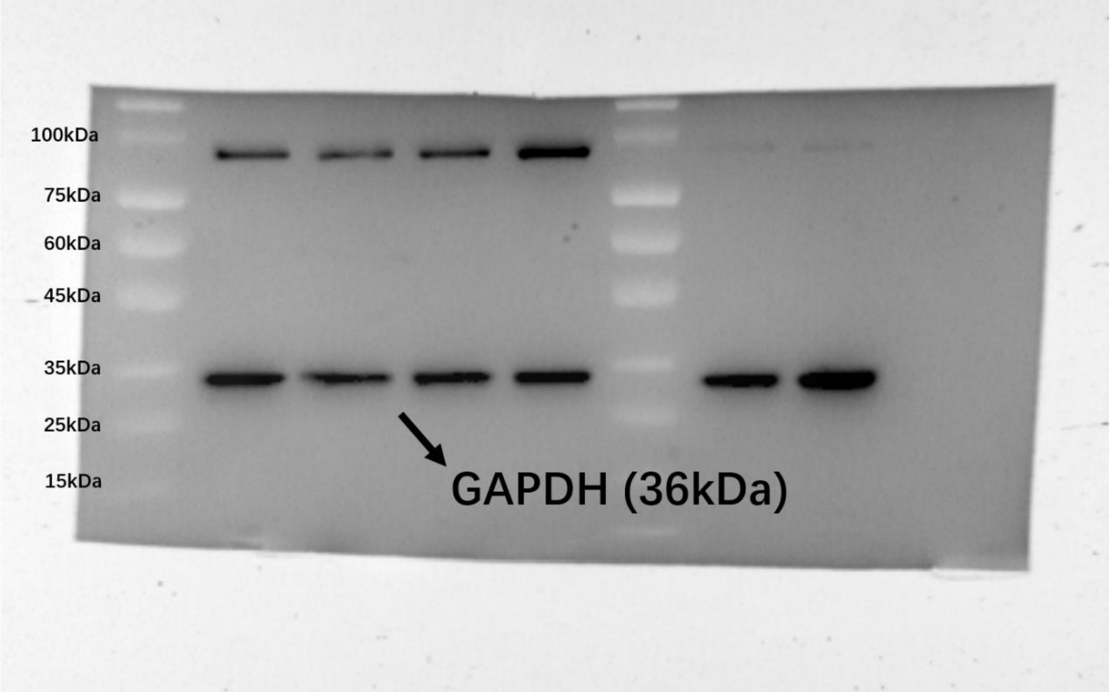
100kDa
75kDa
60kDa
45kDa
35kDa
25kDa
15kDa
GAPDH (36kDa)
